# Dopaminergic axon guidance: which makes what?

**DOI:** 10.3389/fncel.2012.00032

**Published:** 2012-07-31

**Authors:** Laetitia Prestoz, Mohamed Jaber, Afsaneh Gaillard

**Affiliations:** Experimental and Clinical Neurosciences Laboratory, Research Group on Cellular Therapies in Brain Diseases, INSERM U1084, University of PoitiersPoitiers, France.

**Keywords:** mesotelencephalic pathway, development, axon guidance, dopamine, Parkinson's disease, transplantation

## Abstract

Mesotelencephalic pathways in the adult central nervous system have been studied in great detail because of their implication in major physiological functions as well as in psychiatric, neurological, and neurodegenerative diseases. However, the ontogeny of these pathways and the molecular mechanisms that guide dopaminergic axons during embryogenesis have been only recently studied. This line of research is of crucial interest for the repair of lesioned circuits in adulthood following neurodegenerative diseases or common traumatic injuries. For instance, in the adult, the anatomic and functional repair of the nigrostriatal pathway following dopaminergic embryonic neuron transplantation suggests that specific guidance cues exist which govern embryonic fibers outgrowth, and suggests that axons from transplanted embryonic cells are able to respond to theses cues, which then guide them to their final targets. In this review, we first synthesize the work that has been performed in the last few years on developing mesotelencephalic pathways, and summarize the current knowledge on the identity of cellular and molecular signals thought to be involved in establishing mesotelencephalic dopaminergic neuronal connectivity during embryogenesis in the central nervous system of rodents. Then, we review the modulation of expression of these molecular signals in the lesioned adult brain and discuss their potential role in remodeling the mesotelencephalic dopaminergic circuitry, with a particular focus on Parkinson's disease (PD). Identifying guidance molecules involved in the connection of grafted cells may be useful for cellular therapy in Parkinsonian patients, as these molecules may help direct axons from grafted cells along the long distance they have to travel from the substantia nigra to the striatum.

## Introduction

Mesencephalic dopaminergic (mDA) neurons are located in the retrorubral field (RRF; A8 neurons), substantia nigra pars compacta (SNc; A9 neurons), and ventral tegmental area (VTA; A10 neurons) and give rise to ascending axonal projections in the telencephalon. These so-called mesotelencephalic projections are organized into three main pathways: the mesostriatal, mesocortical, and mesolimbic pathways. Axons arising from the VTA and the dorsal part of the SNc and the RRF project to (1) the anteromedial and ventral parts of the striatum (including the nucleus accumbens) and the central nucleus of the amygdala, and (2) the cortex, where they give rise to the mesolimbic and mesocortical pathways. The dorsal and ventral tiers of the SNc and RRF contain dopaminergic neurons that project axons mainly to the dorsolateral (sensorimotor) striatum, and form the mesostriatal pathway or the nigrostriatal pathway, in a restricted sense (Bjorklund and Dunnett, 2007). The latter plays a critical role in the initiation of movement. In humans, the specific loss of SNc dopaminergic neurons is a pathological hallmark of the development and progression of Parkinson's disease (PD). Indeed, dopaminergic cells located in the SNc degenerate, which results in an impaired motor control associated with a dopamine deficit in the striatum. The reasons that this degeneration occurs are not yet fully understood. However, polymorphisms detected in genes coding for axon-guidance molecules are thought to contribute to the pathogenesis of PD, through miswiring of the mesotelencephalic pathway during development, thus increasing the risk of PD (Lesnick et al., 2007). Cell replacement therapy has been investigated in an animal model of PD as a possible means to replace dopaminergic neurons that have been lost. This strategy consists of grafting embryonic dopaminergic neurons in most cases in the striatum, or, less often, in the site of lesion (i.e., in the SN) (Lindvall and Björklund, 2004, 2011; Gaillard and Jaber, 2011). A major challenge in transplant therapies in the SN is to determine to what extent axons of grafted dopaminergic neurons will be able to grow along appropriate pathways to reach their targets. Gaillard et al. (2009) and Thompson et al. (2009) showed that embryonic cells grafted in the lesioned SN of mice resulted in the repair of the lesioned pathway, both anatomically and functionally. These results suggest that guidance cues that specifically govern embryonic fibers outgrowth exist in the adult brain, and that transplanted embryonic cells are able to respond to these cues, which guide them to their final target. Understanding how dopaminergic axons navigate through their native environment during development may contribute to increasing efficiency of cell therapy for brain diseases. Although the ontogeny of the mesostriatal pathway has been investigated for the past 20 years, interesting new data have recently emerged, that we summarize here.

## Establishment of the mesotelecencephalic pathway during embryogenesis

Sonic hedgehog (Shh) signaling triggers the development of dopaminergic neurons from E10 onwards from the ventral midline region (Hynes et al., 1995; Blaess et al., 2006) of the ventricular zone of the rhombencephalic isthmus. These neurons then migrate along the radial glia up to the ventral mesencephalon (VM) (Altman and Bayer, 1981; Specht et al., 1981a,b; Marchand and Poirier, 1983; Smidt and Burbach, 2007; Tang et al., 2009). Proper Wingless-type (Wnt) signaling is required for the correct cell body orientation of mDA neurons, which is disrupted in mice mutant for the planar cell polarity (PCP) receptor *Frizzled3* (Fenstermaker et al., 2010). mDA neurons are immunoreactive for tyrosine hydroxylase (TH) at E12 (Specht et al., 1981a,b) and for dopamine at E14 (Voorn et al., 1988). From E11.5 onwards, mDA neurons extend their axons along the dorsoventral and the anteroposterior axis to reach their telencephalic targets. During this long path to the rostral part of the brain, directed mDA axon growth is dependent on regional specification and patterning within the mesencephalon, diencephalon, and telencephalon. Three main steps are then crucial for axon guidance during mouse embryogenesis. First, from E11.5 to E13.5, the axons of mDA neurons of the mesencephalon extend dorsally from the ventrocaudal region of the midbrain, and then turn rostrally. Second, at E13.5, these axons navigate longitudinally through the midbrain and the diencephalon to form the medial forebrain bundle (MFB). Third, from E14.5 to E18.5, they reach the telencephalon, and more particularly the region of the forebrain that gives rise to the striatum, and innervate the limbic system and the neocortex (Specht et al., 1981a,b; Voorn et al., 1988).

### Axon guidance in the mesencephalon

**Figure 1:**
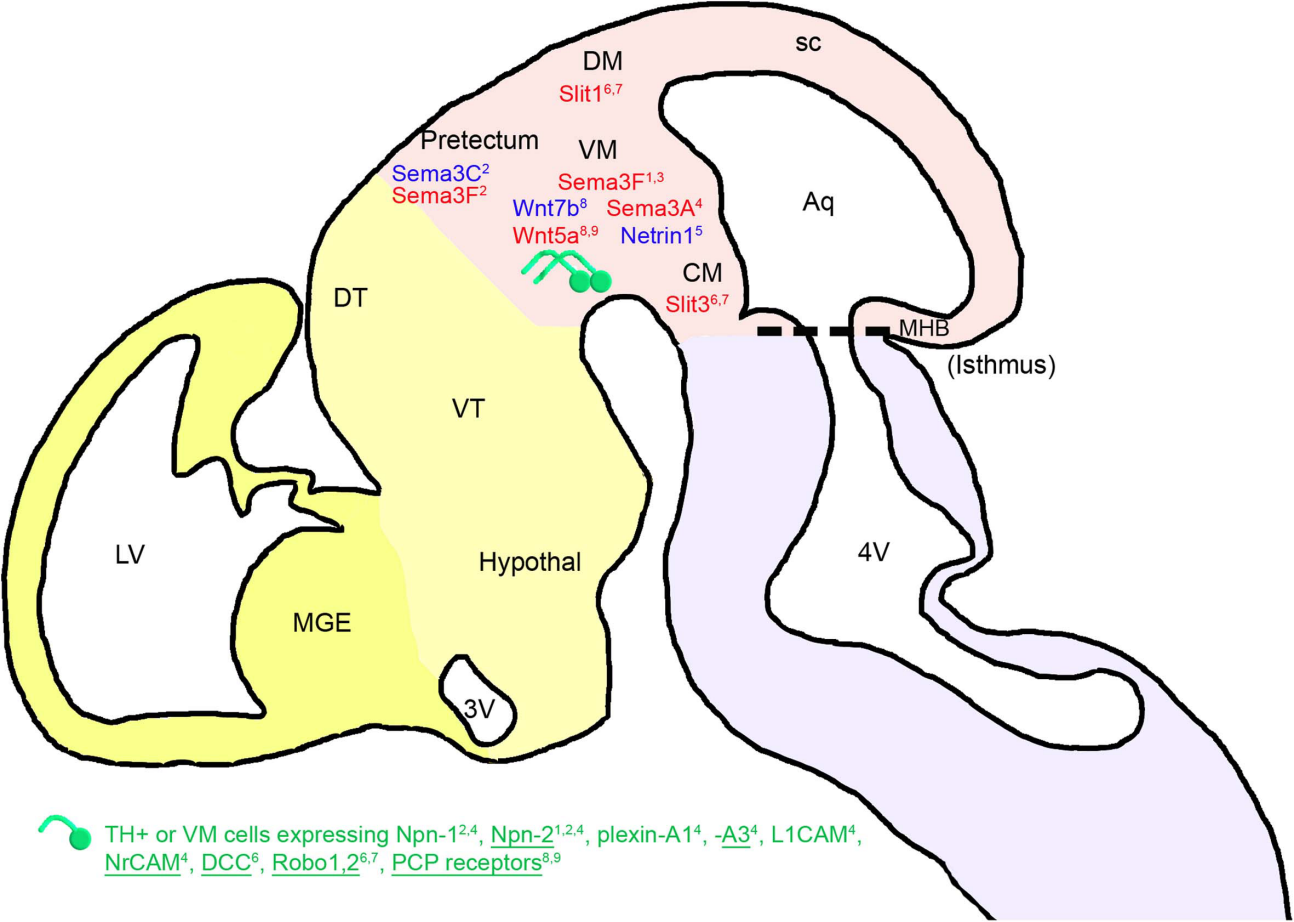
**Mesencephalic guidance: expression of repulsive (in red) and attractive (in blue) guidance cues in the environment of mDA somas and axons between E11.5 and E13.5 in mice.** mDA axons (in green) navigate in a ventrorostral direction. The expression of guidance molecule receptors in these cells is reported: receptors expressed in all or most of the midbrain TH-expressing cells (TH+) are underlined, whereas receptors expressed in a small fraction of TH-expressing neurons are not. The expression of these receptors has been determined either at the mRNA or the protein level, for one or several stages of development between E11.5 and E13.5 (see details in the text). FGF8 has an indirect repulsive action on mDA axons through the activation of Sema3F expression. The cephalic vesicles telencephalon, diencephalon, mesencephalon, and rhombencephalon are delimited in yellow, beige, pink, and purple, respectively. Aq, aqueduct; CM, caudal mesencephalon; DM, dorsal mesencephalon; DT, dorsal thalamus; Hypothal, hypothalamus; LV, lateral ventricle; MGE, medial ganglionic eminence; MHB, midbrain-hindbrain boundary; sc, superior colliculus; VM, ventral mesencephalon; VT, ventral thalamus; 3V, third ventricle; 4V, fourth ventricle. ^1^Yamauchi et al., 2009; ^2^Hernandez-Montiel et al., 2008; ^3^Kolk et al., 2009; ^4^Torre et al., 2010; ^5^Vitalis et al., 2000; ^6^Lin et al., 2005; ^7^Marillat et al., 2002; ^8^Fenstermaker et al., 2010; ^9^Blakely et al., 2011.

After localizing in the VM at E11.5, mDA neurons start their axonal growth dorsally and rostrally, away from the caudal and dorsal mesencephalon (CM and DM) (Figure 1). Nakamura et al. (2000), and then Gates et al. (2004), supported the notion that short-range cues in the midbrain directed the mDA axons rostrally. At the time, the guidance cues involved could not be identified, but the authors showed that removing the diencephalon or the isthmus did not affect the rostrally directed growth of mDA axons. They concluded that the rostral orientation of the mDA fibers was not due to the action of diffusible molecules secreted by the diencephalon. Moreover, considering that mDA neurons localize near the midbrain-hindbrain boundary (MHB), the authors suggested that molecules under the control of the organizing activity of the isthmus, such as ephrin-A2 and ephrin-A5, could control the polarity of axon growth along the rostrocaudal axis through their repulsive activity.

Later, Yamauchi et al. (2009) showed that a signaling center located at the MHB regulates the rostrally directed growth of mDA axons in rats during early development (at E10–E13). They reported that beads soaked with fibroblast growth factor 8 (FGF8), a signaling molecule that mediates the patterning activities of the MHB, repel mDA axons that extend through the diencephalon. The authors suggested that this repulsion could be mediated by semaphorin 3F (Sema3F) because (1) FGF8-soaked beads induced an increase in the expression of Sema3F, (2) Sema3F expression in the midbrain was abolished by a tyrosine kinase inhibitor of an FGF receptor, and (3) mDA axonal growth was inhibited by Sema3F. Furthermore, mDA axons expressed a Sema3F receptor, neuropilin-2 (Npn-2), and the removal of Npn-2 by gene targeting resulted in the aberrant caudal growth of mDA axons. Thus, these results indicate that the MHB signaling center could regulate the growth polarity of mDA axons along the rostrocaudal axis by inducing Sema3F activity. Similarly, the rostrocaudal gradient of Sema3F in the VM may contribute to the rostral orientation of mDA axons by repulsing them rostrally, as suggested by Kolk et al. (2009). Sema3F and Sema3C, which are also expressed in the pretectum, induce a repulsive and an attractive effect on mDA axons, respectively. The pretectal attraction is partially dependent on the interaction with Npn-1 and Npn-2, both expressed in a subset of mDA axons and somas (Hernandez-Montiel et al., 2008). Moreover, Sema3A is detected at E13.5 in the rat VM, and the expression of its receptors and co-receptors Npn-1, Npn-2, plexin-A1, plexin-A3, L1CAM, and NrCAM (Torre et al., 2010) in mDA neurons may also induce, by repulsion, the guidance of mDA axons. More precisely, Sema3A receptors and co-receptors are differentially expressed in mDA cells. Indeed, Npn-1, L1CAM, and plexin-A1 are weakly expressed in a small fraction of TH-expressing neurons in the VM, whereas Npn-1 is strongly expressed in a large number of non-dopaminergic neurons. Npn-2, NrCAM, and plexin-A3 are found in both neurons expressing TH and not expressing TH, and plexin-A3 is more particularly found in a large fraction of TH-expressing neurons (Torre et al., 2010). Plexin-A1 is found in a low number of TH-positive axons in the MFB (Torre et al., 2010), whereas Npn-2 is found on a subset of mDA axons at E13.5 in the rat (Yamauchi et al., 2009). Thus, it is important to consider that different subpopulations of mDA neurons could differentially respond to their environment. Other diffusible guidance molecules expressed in the VM, such as netrin-1, participate in the ventral orientation of mDA axons that express the DCC receptor in rat embryos at E14 (Lin et al., 2005), in a chemorepellent and/or chemoattractive manner (Vitalis et al., 2000).

In addition, Robo1 and Robo2 mRNAs and proteins are differentially expressed during rat embryonic development (from E15 to E20) in the SN and in the VTA. The Robo1 protein is expressed in both calbindin- and Girk2-expressing cells in the VM of rat embryos at E14, while the Robo2 protein is exclusively found in the calbindin-expressing subpopulation (Lin et al., 2005). This suggests a differential guidance between the two subpopulations of mDA neurons. A repulsive interaction between Robo1 and Robo2 receptors expressed by mDA neurons and their ligands (Slit-1 and Slit-3) present in the DM and CM contributes to the rostrocaudal reorientation of mDA axons (Marillat et al., 2002; Lin et al., 2005). Fenstermaker et al. (2010) recently revealed the role of Wnt and PCP receptor proteins in the anteroposterior guidance of mDA axon projections. Frizzled3, Celsr3, and Vangl2 PCP receptors are widely expressed in the midbrain region and in all TH-expressing mDA neurons, as shown by co-immunostaining against PCP proteins and TH between E11.5 and E14.5 in mouse embryos (Fenstermaker et al., 2010). In addition, Wnt5a is expressed in a high posterior/low anterior gradient, whereas Wnt7b is expressed in a high anterior/low posterior gradient in the midbrain. Wnt5a promotes the retraction and repulsion of mDA axons partly through Frizzled receptors, as shown by *in vitro* functional assays (Blakely et al., 2011), whereas Wnt7b has an attractive effect. In PCP signaling mutants, mDA axons are severely misguided along the anteroposterior axis. Indeed, instead of being rostrally oriented, as shown in wild-type mice, mDA axons of *Frizzled3*, *Celsr3*, and *Vangl2 (Lp/Lp)* mutant mice display aberrant dorsal and caudal projections (from E12.5 until E17.5).

### Axon guidance in the diencephalon

**Figure 2:**
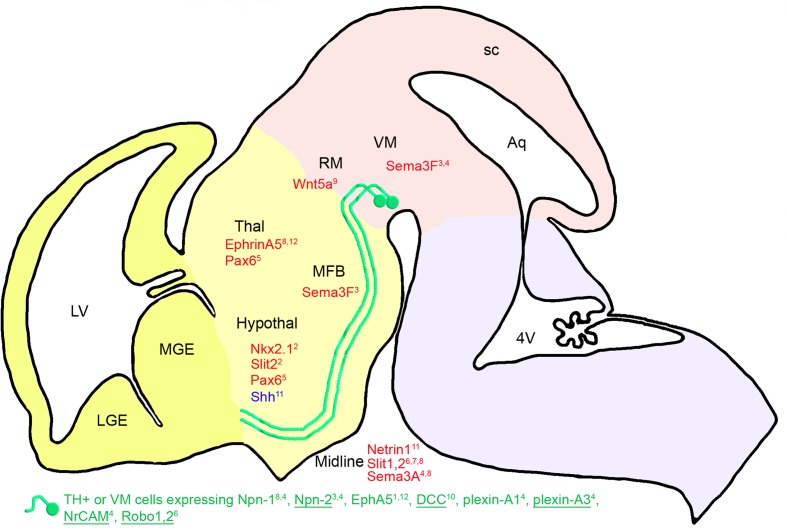
**Diencephalic guidance: expression of repulsive (in red) and attractive (in blue) guidance cues in the environment of mDA somas and axons at E13.5 in mice.** mDA axons (in green) fasciculate in a ventrorostral direction to form the MFB. mDA axons respond to environmental cues through specific receptors (in green). Receptors expressed in all or most of the midbrain TH-expressing cells (TH+) are underlined, whereas receptors expressed in a small fraction of TH-expressing neurons are not. The expression of these receptors has been determined either at the mRNA or the protein level around E13.5 (see details in the text). Nkx2.1 has an indirect repulsive action on mDA axons through the activation of Slit-2 expression. Pax6 allows the mDA fibers to navigate ventrally through the expression of a repulsive action of netrin-1. The cephalic vesicles telencephalon, diencephalon, mesencephalon, and rhombencephalon are delimited in yellow, beige, pink, and purple, respectively. Aq, aqueduct; Hypothal, hypothalamus; LGE, lateral ganglionic eminence; LV, lateral ventricle; MFB, medial forebrain bundle; MGE, medial ganglionic eminence; RM, rostral mesencephalon; sc, superior colliculus; Thal, thalamus; VM, ventral mesencephalon; 4V, fourth ventricle. ^1^Deschamps et al., 2009; ^2^Marín et al., 2002; ^3^Kolk et al., 2009; ^4^Torre et al., 2010; ^5^Vitalis et al., 2000; ^6^Lin et al., 2005; ^7^Dugan et al., 2011; ^8^Kawano et al., 2003; ^9^Blakely et al., 2011; ^10^Xu et al., 2010; ^11^Hammond et al., 2009; ^12^Deschamps et al., 2010.

Once the rostrally oriented growth begins at E13.5, mDA axons become highly restricted to a narrow path. This narrow path appears to result from multiple signals that keep axons from diverging ventrally or dorsally (Figure 2).

In 1992, the presence of adhesive molecules was proposed to explain the fasciculation of mDA neurons in the MFB (Shults et al., 1992). Indeed, the nerve growth factor-inducible large external (NILE) protein is mostly present between E13 and E20, in fasciculated mDA axons coursing rostrally from the mesencephalon. The authors hypothesized that NILE found on the surface of pioneer axons could define a pathway with adhesive cues for these axons, as it had been shown for L1/NgCAM, which promotes the elongation of axons from embryonic mDA neurons *in vitro* (Shults et al., 1992).

Since then, several other diffusible guidance molecules playing a role in maintaining the MFB fasciculation have been described. Indeed, Sema3F expressed in the VM may participate to tighten this tract *via* its repulsive interaction with Npn-2 expressed by mDA neurons (Kolk et al., 2009; Torre et al., 2010). Similarly, the repulsive interaction between Sema3A, expressed in the midline, and its receptors Npn-1, Npn-2, plexin-A1, and plexin-A3, expressed by mDA neurons, could help maintain these fibers in an ipsilateral trajectory (Kawano et al., 2003) Double immunostaining in rat embryos at E13.5 and in VM cultures showed that Npn-2, NrCAM, and plexin-A3 are expressed in a large subset of TH-expressing neurons (Kolk et al., 2009; Torre et al., 2010), whereas single immunostaining for Npn-1 showed that the MFB fibers are faintly immunoreactive for this receptor in mouse embryos at E12.5 (Kawano et al., 2003). This suggests that Sema3A may principally acts on mDA axons through its interaction with Npn-2, NrCAM, and plexin-A3.

The presence of diffusible repulsive molecules, such as Slit-1 and Slit-2, in the midline of mouse embryos at E13.5 (Bagri et al., 2002; Marín et al., 2002) prevents MFB fibers from crossing *via* their interactions with Robo1 and Robo2 expressed in subpopulations of mDA neurons (Lin et al., 2005). Indeed, mDA fibers in the MFB of Slit-2 mutants are ventrally displaced as they course through the diencephalon. In Slit-1-Slit-2 double mutants, the MFB is split into two components and numerous fibers descend ventrally into the hypothalamus, toward the midline (Bagri et al., 2002). Dugan et al. (2011) confirmed that the Slit proteins contribute to guiding the mDA projections through the diencephalon. Indeed, mDA axons make significant pathfinding errors in Slit-1/2 and Robo1/2 double knockout mice, such as spreading out in the diencephalon to form a wider tract. The wider tract results from a combination of the invasion of the ventral midline, consistent with Slit repulsion, and a dorsal expansion of axons, away from the ventral midline. Aberrant dorsal trajectories are prominent in Robo1 and Robo1/2 knockout mice, suggesting that Robo proteins may promote mDA axon fasciculation into tightly organized tracts, independently of Slit proteins. Moreover, mDA axons are not pioneers, but project into an environment where they likely interact and possibly fasciculate with pre-existing tracts. As earlier tracts may also be dependent on Slit/Robo guidance (Marion et al., 2005; Tsuchiya et al., 2009), mDA projections errors may be a secondary consequence of the earlier tract errors.

Short-range cues may also participate in maintaining the MFB fasciculation. Indeed, ephrin-A5 expression in the thalamus (Deschamps et al., 2009, 2010) may maintain mDA fibers in a ventral position, on one hand, through its repulsive interaction with EphA5 expressed in the mDA neurons (Deschamps et al., 2009), and on the other hand, *via* its positive action on axon growth (Cooper et al., 2009). Indeed, it has been shown *in vitro* that neurons originating from the VM grow on thalamic explants without being connected to it (Gates et al., 2004).

Marín et al.' (2002) studies on Nkx2.1 mutant mice revealed the importance of ephrin, semaphorin and Slit molecules for mDA axon guidance. Indeed, these authors found that mDA fibers abnormally converge toward the rostral midline in the diencephalon (hypothalamus) in the absence of Nkx2.1. In this mutant, the expression of ephrin-A5, EphB2, EphB3, Sema3C, and Slit-2 is downregulated, whereas the expression of Sema3A, Sema3F, and Slit-1 is upregulated. Slit-2 expression is absent from the hypothalamus, where it normally contributes to repel mDA fibers from the midline.

In addition, Shh, which is expressed in the ventral-medial midbrain and hypothalamus during mDA axonal outgrowth, acts as a local guidance cue for medially projecting mDA axons. Indeed, in midbrain explants, dopaminergic projections are attracted to an Shh source. In addition, the most medial dopaminergic projections are deficient when Shh signaling is inactivated during late neuronal development in mice (Hammond et al., 2009). These results suggest that medial and lateral mDA neuron populations have a differential sensitivity to Shh chemoattraction.

Although mDA neurons do not express Pax6 at E13.5 (Vitalis et al., 2000), mice lacking Pax6 display an altered pathfinding of mDA projections. Thus, instead of following the route of the MFB ventrally, most of the mDA axons are deflected dorsorostrally at the pretectal-dorsal thalamic transition zone and in the dorsal thalamic alar plate, migrating away from the abnormally expanded netrin-1 expression. Moreover, Xu et al. (2010) detected an aberrant dopaminergic ventral commissure descending from the MFB and crossing the midline at the level of the hypothalamus in *DCC* mutant mice. These data suggest that netrin-1 has a chemorepellent activity on the pathfinding of mDA neurons mediated by the DCC receptor, which is expressed in a large number of TH-expressing cells, as shown by immunohistochemistry in mouse embryos at E14 (Xu et al., 2010).

More recently, Blakely et al. (2011) showed that Wnt5a mutant mice display an abnormal fasciculation of mDA axons in the MFB. Wnt5a is normally expressed in the vicinity of the mesostriatal pathway during development and acts as a chemorepellent for mDA neurites as shown in VM explants *in vitro*.

### Axon guidance in the telencephalon

**Figure 3:**
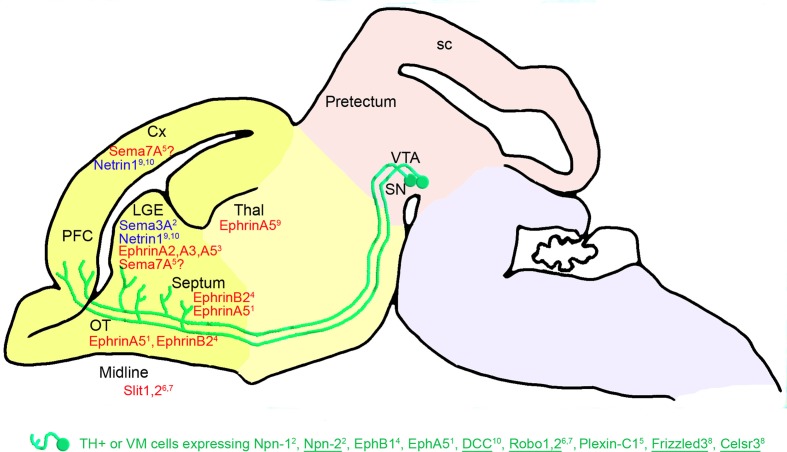
**Telencephalic guidance: expression of repulsive (in red) and attractive (in blue) guidance cues in the environment of mDA somas and axons between E14.5 and E18.5 in mice.** mDA axons (in green) connect to the telencephalic regions through specific receptors (in green). The expression of these receptors has been detected in the mDA cells in the VM using double immunostaining against TH and protein receptors. Receptors expressed in all or most of the midbrain TH-expressing (TH+) cells are underlined, whereas receptors expressed in a small fraction of TH-expressing cells are not. EphB1 has been detected at the mRNA level in the VM. The repulsive effect of Sema7A on mDA axons remains to be determined^5^. The cephalic vesicles telencephalon, diencephalon, mesencephalon, and rhombencephalon are delimited in yellow, beige, pink, and purple, respectively. Cx, cortex; LGE, lateral ganglionic eminence; SN, substantia nigra; OT, olfactory tract; PFC, prefrontal cortex; sc, superior colliculus; Thal, thalamus; VTA, ventral tegmental area. ^1^Deschamps et al., 2009; ^2^Hernandez-Montiel et al., 2008; ^3^Janis et al., 1999; ^4^Yue et al., 1999; ^5^Pasterkamp et al., 2007; ^6^Lin et al., 2005; ^7^Marillat et al., 2002; ^8^Fenstermaker et al., 2010; ^9^Hamasaki et al., 2001; ^10^Xu et al., 2010.

After their navigation in the MFB, mDA projections invade the striatum in a ventrodorsal direction between E14.5 and birth (Figure 3). A high rostral and ventral ephrin-A5 expression was detected in the striatum (Deschamps et al., 2009), suggesting that ephrin-A5 may repel mDA projections (as shown *in vitro*) toward the domains with low dorsal and caudal ephrin-A5 expression. This ephrin-A5 expression gradient may help the mDA axons to spread within the striatum: low-responding ephrin-A5 mDA axons may be preferentially connected in the ventral part of the striatum, whereas high-responding ephrin-A5 mDA axons may be repelled toward the dorsal part of the striatum. Moreover, in the most ventral part of the telencephalon, mDA axons not responding to ephrin-A5 may grow to connect cortical regions. This neural map specification through gradients is a well-known mechanism used by ephrins. Ephrins regulate axon growth allowing axons to terminate at a neutral or optimum point in the gradient, as extensively shown for the retinotectal and thalamocortical systems (Flanagan, 2006). This mechanism could explain the graded distribution of dopaminergic projections in the striatum during development and the extinction of ephrin-A5 expression when mDA axons connect to the entire striatum at P7. Moreover, this model was supported by the work of Sieber et al. (2004), who showed that the disruption of EphA-ephrin-A interactions resulted in the mistargeting of a fraction of SN dopaminergic projections, leading to a reduced number of dopaminergic projections in the dorsolateral striatum. Indeed, according to this model, a lack of ephrin-A–EphA signaling may induce fewer dopaminergic projections connecting to the dorsal part of the striatum, since they would not be repelled from the ventral part. These axons would grow more ventrally instead of turning dorsally into the striatum, and would then be misrouted in the absence of ephrin-A–EphA signaling.

Other ephrins/Ephs may also be implicated in the establishment of these mesostriatal connections, such as EphB1, the mRNA of which is differentially expressed in the VM (Yue et al., 1999). Indeed, EphB1 is more intensively expressed in dopaminergic neurons of the SNc than in dopaminergic neurons of the VTA region in newborn mice. Its repulsive interaction with ephrin-B2 strongly expressed in the ventral striatum may contribute to the navigation of the nigrostriatal fibers toward the dorsal part of the striatum (Yue et al., 1999; Hu et al., 2004). Later in the postnatal development, dopamine activity has been shown to regulate the developmental expression of EphB1 (Halladay et al., 2000). Finally, the presence of ephrin-A2, -A3, and -A5, differentially expressed in the striatal compartments, matrix and striosome, suggests their implication in the formation and stabilization of mesostriatal connections (Janis et al., 1999).

In addition, Marillat et al. (2002) suggested that mDA axons could be guided through a Robo-Slit interaction toward their telencephalic targets. Indeed, they showed that rat embryonic mDA cells from the SNc express Robo1 and Robo2 mRNAs, and that VTA dopaminergic cells mainly express Robo1 mRNA. In the telencephalon, Slit-1 mRNA is expressed in the caudate putamen, whereas Slit-1 and Slit-2 mRNAs are expressed in the septum. Bagri et al. (2002) studied the distribution of mDA fibers in *Slit* mutant mice. Although many dopaminergic fibers entered the telencephalon normally in *Slit-1-Slit-2* mutants, a significant percentage abnormally crossed the midline in the basal telencephalon. These defects were readily apparent at E14.5, suggesting that loss of Slit function affects the development of dopaminergic systems as they course rostrally into the forebrain. Slit-1 and Slit-2 prevent the growth of dopaminergic axons into ventral domains of the forebrain. In addition, Slit-1 inhibits the axon growth of midbrain dopaminergic neurons *in vitro* (Lin et al., 2005).

At E15.5 in the rat, Pasterkamp et al. (2007) showed that neurons in the central part of the VTA express the plexin-C1 receptor and showed that its ligand Sema7A is detected in a subset of SNc neurons, as well as in the striatum and the cortex. This suggests that the interaction between plexin-C1 and Sema7A contributes to the navigation of mDA axons to the telencephalon. Other semaphorin receptors, such as Npn-1, Npn-2, plexin-A1, and plexin-A3, are also expressed in mDA neurons (Hernandez-Montiel et al., 2008; Torre et al., 2010). Npn-1 and Npn-2 are more particularly present in a fraction of mDA neurons and in mDA axons connecting pretectum explants in midbrain-pretectum co-cultures (Hernandez-Montiel et al., 2008). From a functional perspective, the interaction between Sema3F and Npn-2 mediates a chemoattraction that is required to orient mDA axon projections to the cortical plate of the medial prefrontal cortex (Kolk et al., 2009). When expressed in HEK293 cell aggregates, Sema3A enhances axon growth. In addition, co-culture explants from the striatum and the VM show an attractive effect on mDA neurons. Attraction by the striatum is not affected by the presence of anti-Npn-1 antibodies (Hernandez-Montiel et al., 2008). Overall, these studies support a role for semaphorin signaling in dopaminergic axon pathfinding.

The attractive interaction between netrin-1 and DCC may also play a role in the mDA axon guidance to the striatum and the cortex, through the expression of netrin-1 in these target regions and the expression of DCC in mDA neurons (Hamasaki et al., 2001; Xu et al., 2010). Moreover, UNC5, a netrin-1 receptor, is also expressed in mDA neurons of rat embryos at E18 (Xu et al., 2010). Interestingly, the DCC/UNC5 ratio in mDA neurons varies during development, thus modulating the response of these neurons to netrin-1, and consequently modifying the organization and function of mesocorticolimbic dopaminergic systems (Manitt et al., 2010).

Finally, the marked defects in mDA axon projections found in the telencephalon of mutant mice for the PCP components *Frizzled3* and *Celsr3* support the idea that Wnt signaling may control anteroposterior guidance in the forebrain. Fenstermaker et al. (2010) showed that, in the telencephalon, mDA axons of *Frizzled* mutant mice are displaced ventrally and are located in close vicinity to the optic chiasm. No innervation of synaptic target regions, such as the striatum or medial prefrontal cortex, by mDA axons is found at E14.5 or E17.5. In *Celsr3* mutant mice, many mDA axons follow aberrant lateral, dorsal, and ventral trajectories, and in contrast to *Frizzled* mutant mice, a small subset of mDA axons reaches, but does not innervate, the striatum at E17.5 (Fenstermaker et al., 2010). Celsr3 is a protocadherin known to be critical for the development of several major axonal bundles in the CNS (Tissir et al., 2005). Thus, Wnt/PCP signaling may be an anteroposterior guidance mechanism that controls the axonal and cellular organization of this mesotelencephalic pathway. However, Wnt5a does not seem to be the main actor involved, as mDA axon projections in *Wnt5a* mutant mice only display a minor and transient posterior projection, suggesting that the lack of Wnt5a signaling is compensated by other Wnt proteins in late development stages (Fenstermaker et al., 2010).

## Axonal guidance cues in the adult mesotelencephalic pathways

### In the intact adult brain

In the intact adult brain, the expression of axonal guidance cues is globally downregulated with a persistent expression confined to regions of high-plasticity (Yamaguchi and Pasquale, 2004). Depending on the nature of the molecules considered, the localization of their expression is either conserved throughout the embryonic, postnatal, and adult life or may totally differ between the embryonic and adult brains.

For instance, short-range cues, such as ephrins, are present at low levels in the adult nigrostriatal system (Goldshmit et al., 2006). Their function is to maintain the neuronal connections and synaptic plasticity (Goldshmit et al., 2006). Ephrin-B2 is expressed in the SN and the VTA, in the amygdala, hypothalamus and, to a lesser extent, in the caudate putamen (Migani et al., 2007). Several Eph receptors are also detected in the adult brain, such as EphA4 in the nucleus accumbens, the hippocampus and the thalamus (Xiao et al., 2006), as well as in the SN and the cortex (Martone et al., 1997; Xiao et al., 2006). EphA5 is expressed in the VTA, the SN, the thalamus, the striatum, and the cortex (Olivieri and Miescher, 1999; Cooper et al., 2009). The constitutive expression of the dominant negative EphA5 receptor containing a disrupted tyrosine kinase domain in the mouse hippocampus and striatum results in altered striatal functioning, and more particularly, in spatial navigation accompanied by a decrease in striatal dopamine (Yue et al., 2002; Halladay et al., 2004). EphB1 is expressed in the nucleus accumbens, in the thalamus and in the matrix compartments of the striatum (Martone et al., 1997; Xiao et al., 2006), whereas EphB2 is strongly expressed in the cortex (Moreno-Flores and Wandosell, 1999).

Some long-range cues are also present in the adult brain, such as the DCC-netrin family, and their expression appears to be similar to that of the developing brain. Indeed, DCC immunoreactivity has been detected in A9 dopaminergic neurons in the SN, and in A10 dopaminergic neurons predominantly located in and around the interfascicular nucleus. Terminal fields selectively labeled with DCC antibodies correspond to known nigrostriatal projections to the dorsolateral striatal patches and dorsomedial shell of the accumbens. The DCC immunoreactivity is also detected in the prefrontal cortex, the septum, the lateral habenula, and the ventral pallidum. This unique distribution of DCC immunoreactivity in adult mDA neurons suggests that netrin-1/DCC signaling could contribute to the plasticity and remodeling of dopaminergic projection pathways (Osborne et al., 2005). Indeed, changes in netrin-1 receptor expression, through the modulation of cAMP levels (Jassen et al., 2006), may play a role in the lasting effects of exposure to amphetamine and other stimulant drugs (Yetnikoff et al., 2007), and may contribute to cognitive deficits associated with drug abuse (Bahi and Dreyer, 2005). Netrin-1's involvement in the rodent mesocorticolimbic dopaminergic system has been recently confirmed by Flores (2011), who described an upregulation of netrin-1 receptors by repeated exposure to stimulant drugs of abuse in dopaminergic somatodendritic regions and a role for netrin-1 in drug-induced behavioral plasticity. Moreover, Manitt et al. (2011) revealed the implication of DCC-mediated netrin-1 signaling in the establishment of medial prefrontal cortex dopaminergic circuitry.

The distribution of secreted semaphorins has been extensively studied in the postnatal and adult hippocampus of rodents. These studies indicate that the expression of transcripts for specific secreted semaphorins and neuropilins persists in a variety of mature neurons (Hirsch et al., 1999; Miyasaki et al., 1999; Giger et al., 2000; Holtmaat et al., 2002; Bagri et al., 2003; Barnes et al., 2003). Their localization indicates that they are likely to function at the level of synapses (Mann et al., 2007). Thus, Sema3A could modulate the morphology and the function of synapses in the adult hippocampus (Bouzioukh et al., 2006). More recently, a study on a putative Sema3F co-receptor, i.e., plexin-A4, showed that plexin-A4 was detected in neurons and fibers throughout the brain and spinal cord, including the neocortex, hippocampus, lateral hypothalamus, red nucleus, facial nucleus, and the mesencephalic trigeminal nucleus. Plexin-A4 antibodies labeled fibers in the lateral septum, nucleus accumbens, several thalamic nuclei, SN pars reticulata, zona incerta, and pontine reticular region, as well as in several cranial nerve nuclei (Gutekunst et al., 2010).

Guidance molecules from the Slit/Robo family are also widely expressed in the adult brain, as reported by Marillat et al. (2002). For example, Robo1 and Robo2 are expressed in the caudate putamen, nucleus accumbens, thalamus, hypothalamus and SN, and Robo1 is exclusively expressed in the VTA. Slit-1 is expressed in the caudate putamen and the VTA, while Slit-1 and Slit-2 are expressed in the thalamus and the hypothalamus, as well as in the SN (Marillat et al., 2002).

Finally, Shh and Wnts are also present in the adult brain, where Shh receptors are expressed in a few areas, such as the hippocampus and the superior colliculus (Charytoniuk et al., 2002). The Wnt signaling pathway seems to be involved in adult neurogenesis (Malaterre et al., 2007).

### In the lesioned adult brain

After a lesion, the expression of ephrins, semaphorins, netrins, and Slits is modulated at the lesioned site in the adult brain, and has been shown to prevent an endogenous regeneration in most cases. Indeed, ephrin-B2-EphB2 and EphA4 participate in the formation of the glial scar after spinal cord injury (Bundesen et al., 2003; Fabes et al., 2006) and prevent the regeneration of corticospinal tract axons, whereas EphA4 blockers promote axonal regeneration and functional recovery after spinal cord lesion in mice (Goldshmit et al., 2011). Moreover, the upregulation of EphA4 in astrocytes seems to mediate astrocytic gliosis after cortical lesion in the Marmoset Monkey (Goldshmit and Bourne, 2010). Ephrin-B1 expression is also upregulated in reactive astrocytes present in the denervated hippocampal region, whereas Eph receptors are expressed in sprouting axons (Wang et al., 2005).

Long-range cues, such as Sema3A, may also prevent the regeneration of lesioned axons through a chemorepulsive signal resulting from its interaction in scar tissue with Npn-1 expressed in regenerating olfactory axons (Pasterkamp et al., 1998). In the mouse spinal cord and cerebellum, netrin-1, Slit-1, and Slit-3 are expressed at the lesion site in macrophages and fibroblasts, where they may contribute to the regenerative failure of axons in the adult CNS, by inhibiting axon outgrowth or by participating in the formation of the CNS scar (Wehrle et al., 2005).

Lesioning of the mesotelencephalic pathway using 6-hydroxydopamine injections in the striatum results in an increase of Sema3A expression in striatal astrocytes one week after the injection (Yasuhara et al., 2004). Thus, Sema3A could play a role in the induction of cell death in dopaminergic neurons.

Overall, these repulsive molecular interactions after a lesion may prevent an endogenous repair at the lesion site and, in some cases, may contribute to the death of mDA neurons. However, a repulsive activity may also be useful, if expressed outside of the lesioned site, to maintain axons (either endogenously regenerated axons or axons arising from grafted cells) on their pathway to their final target. Then, it would be interesting to study whether the chemical lesioning of the mesotelencephalic pathways could induce a modulation of the expression of axon guidance cues in the adjacent regions.

### In the grafted adult brain

Several studies have investigated the potential of cell therapy in animal models of PD. All these models are based on the induction of chemical lesions of the nigrostriatal dopaminergic pathway by injecting a toxin either into the SN or the striatum, or more rarely into the MFB. The grafted cells could be rodent or human embryonic stem (ES) cell-derived dopaminergic neurons, rodent or human mesencephalic fetal cells or induced pluripotent stem (iPS) cells (for review, see Gaillard and Jaber, 2011).

Zhou and Chiang (1995) first described a trophic effect of excitochemicals injected after a lesion of the MFB, serving as effective axonal guidance for fetal neurons to innervate distal brain regions. Moreover, embryonic nigral transplants implanted in the striatum are capable of promoting growth and providing guidance to axons arising from a dopaminergic graft placed homotopically in the VM, resulting in dopaminergic nigrostriatal reinnervation (Mendez et al., 1996). Double grafts in the striatum and SN can also reconnect the striato-nigro-striatal circuitry (Mendez and Hong, 1997). Grafted cell lines, such as the human teratocarcinoma cell line (Baker and Mendez, 2005), also allow for a reconstruction of the dopamine-denervated nigrostriatal pathway. In 2001, the study of the therapeutic potential of grafted cells crossed an important step with the generation of transgenic mice expressing green fluorescent protein (GFP) under the control of the rat TH gene promoter. GFP cells isolated using fluorescence-activated cell sorting were transplanted into a rat model of PD. Surviving cells innervated the host striatum, resulting in a recovery of Parkinsonian behavioral defects (Sawamoto et al., 2001). This strategy is very interesting, because it allows grafted cells expressing GFP to be visualized in the host brain. These advances lead to the publication of a series of studies describing the reconstruction of the nigrostriatal pathway after transplantation. Indeed, fetal dopaminergic neurons implanted into the SN of adult mice are capable of substantially reconnecting the nigrostriatal pathway with an outgrowth pattern that matches the anatomy of the endogenous system (Gaillard et al., 2009; Thompson et al., 2009). More precisely, Thompson et al. (2005) showed that, in VM grafts, the dopaminergic innervation of the striatum is derived almost exclusively from Girk2-positive SN cells, whereas calbindin-positive VTA neurons project to the frontal cortex and probably also other forebrain areas. Furthermore, the A9 component of intrastriatal grafts is critical for recovery, as shown through tests of motor performance in a rodent model of PD (Grealish et al., 2010). This suggests that specific guidance cues exist in the host brain that can differentially guide the mDA axon subpopulations to their respective, appropriate targets. Moreover, grafted cells seem to be able to respond to these environmental cues. Indeed, rodent mesencephalic fetal cells are known to express receptors to guidance cues, such as neuropilins (Hernandez-Montiel et al., 2008; Kolk et al., 2009; Yamauchi et al., 2009), plexins (Torre et al., 2010), cell adhesion molecules (Torre et al., 2010), DCC (Lin et al., 2005), Robos (Marillat et al., 2002; Lin et al., 2005), and PCP receptors (Fenstermaker et al., 2010; Blakely et al., 2011) (see details in the first part of this review). However, whether the expression of specific guidance cues is modulated in the host brain after transplantation remains to be determined.

In spite of their therapeutic potential, the use of fetal cells is not without significant technical, ethical, political, and logistical issues that limit their generalized used in cell therapies. ES cell-derived dopaminergic neurons are more widely available and provide a great hope for cell replacement therapy in PD (Kriks et al., 2011). Like fetal dopaminergic neurons, these cells express the netrin receptor DCC and the Slit receptor Robo. Slit-2 repels fetal dopaminergic neurites (Lin et al., 2005), as well as mouse and human ES-derived DA axons (Lin and Isacson, 2006; Cord et al., 2010). However, while netrin-1, Slit-1, and Slit-3 molecules can guide fetal dopaminergic axons (Lin et al., 2005; Lin and Isacson, 2006), no directed neurite outgrowth was observed in the co-cultures of ES cell-derived dopaminergic neurons with netrin-1, Slit-1, and Slit-3-producing cells (Lin and Isacson, 2006). These findings suggest that ES cell-derived dopaminergic neurons generated by current protocols can respond to guidance cues *in vitro* in a similar manner to fetal cells. They also indicate that these cells exhibit distinct responses that could be explained by developmental differences generated by current *in vitro* methods of cell patterning or conditioning during ES cell differentiation (Lin and Isacson, 2006). Moreover, human ES-derived dopaminergic neurons attain peak responsiveness to these cues over time in culture, confirming that the timing of dopaminergic transplant is an important variable for future studies using human ES-derived neurons in therapy. Other studies (Tamariz et al., 2010) also showed that similar proportions of ES cell-derived dopaminergic neurons and fetal cells from embryonic VM express the semaphorin receptors Npn-1 and Npn-2. Furthermore, the axons of both populations respond very similarly to semaphorin exposure. Indeed, Sema3A increases axon length, and Sema3C attracts axons and increases their length. These effects are mediated by neuropilins, since the addition of blocking antibodies against these proteins reduced the effects on axonal growth and attraction. The phenotypic similarities between ES cell-derived dopaminergic neurons and dopaminergic neurons from embryonic VM suggest that Sema3A and Sema3C may be used to guide axons of grafted ES cell-derived dopaminergic neurons in therapeutic protocols for PD (Tamariz et al., 2010). Sema3A and Sema3C could be delivered at the target site to attract cell-transplanted axons using a viral vector-mediated strategy, as used for the expression of neurturin in the striatum of nonhuman primates (Herzog et al., 2009), and more commonly, for neurotrophic factors (for review Rangasamy et al., 2010). However, as early stages of PD in humans leave a significant proportion of the nigrostriatal projections intact, which may serve as a substrate for regeneration and functional recovery (Bjorklund et al., 1997), it is crucial to consider the possible duality of action of exogenous guidance molecules on these regenerating axons and on the transplanted-cell axons. Indeed, axonal growth and sprouting of fibers in the CNS is partially inhibited by guidance molecules, such as canonical axon guidance molecules (e.g., semaphorins, ephrins, netrins), prototypic myelin inhibitors (Nogo, MAG, and OMgp), and chondroitin sulfate proteoglycans (lecticans, NG2) (Giger et al., 2010). Sema3A is more particularly known to inhibit the regeneration of CNS axons (Pasterkamp and Giger, 2009), although whereas it has a positive, attractive effect on embryonic and ES-derived dopaminergic neurons, as described above (Tamariz et al., 2010).

Apart from acting on the expression of guidance cues localized in the environment of the pathway, another way to improve the transplantation efficacy could be to modulate the expression of single or combined guidance cue receptors in the cells to be transplanted. Enhancing or repressing their response to environmental cues may be achieved using gene therapy, as it has been previously done to replace enzymes involved in the dopamine metabolism (for review, see Bjorklund et al., 2010). However, modifying the expression of guidance molecule receptors before transplantation would induce a modification of the axon guidance all along the nigrostriatal pathway, from the initial site of transplantation to the target site. This is an important issue, because environmental cues do not direct the mDA axons in the same manner all along their growth to the striatum. For example, mesoprefrontal dopaminergic axons change their responsiveness to Sema3F through the Npn-2 receptor, from repulsion to attraction through their course to the cortex (Kolk et al., 2009).

Ultimately, stem cell-derived dopaminergic neurons should become more widely available, in large part thanks to recent technological advances that could allow for dopaminergic neurons to be obtained from somatic cells of patients. Indeed, Wernig et al. (2008) provided data suggesting recovery in rat models of PD using iPS cells. Thus, because of their plasticity and ability to undergo directed differentiation, iPS cells are promising candidates to replace dopaminergic cells and integrate themselves synaptically into the recipient brain, thus providing a possible alternative for the treatment of PD (Chen et al., 2011). The expression of guidance cue receptors in iPS cells and the functional effects of the molecular environment on axon guidance remain to be determined. Indeed, the response to the known embryonic dopaminergic axon guidance systems of the cells to be transplanted *in vitro*, as well as after grafting, must be analyzed. This is of paramount importance for the development of efficient cell replacement strategies in PD, even if a partial restoration of dopamine levels is sufficient to restore function in PD models (Gaillard et al., 2009).

### In parkinsonian patients

As early as 1997, experimental studies inferred that a genetic variability in the mDA axon guidance systems could contribute to PD (Livesey and Hunt, 1997). Indeed, gene polymorphisms in the mDA axon guidance system could result in an aberrant trajectory of the ascending dopaminergic pathway during embryonic brain development, and could then cause a congenital deficiency in nigrostriatal dopamine. These developmental anatomic asymmetries in nigrostriatal innervation could account for motor asymmetries later seen in patients (Djaldetti et al., 2006). Moreover, individuals with these polymorphisms could be at greater risk for PD and at an earlier age (Lesnick et al., 2007), as previously described in patients with Tourette's syndrome, which is associated with a rare sequence variation in a single axonal-dendritic development gene (Abelson et al., 2005).

Several polymorphisms have been described in genes coding for axon guidance cues. For instance, the Semaphorin5A gene (*SEMA5A*) was shown to contain the single nucleotide polymorphism (SNP) most significantly associated with PD susceptibility (Maraganore et al., 2005). Although Li et al. (2008) challenged the robustness of the panel of genetic markers used to predict PD risk and the mDA axon guidance system implication in PD genetics, other SNPs have been recently reported as possible susceptibility factors for PD (Kim et al., 2011). Indeed, SNPs in the *DCC*, *calcium binding protein P22* (*CHP*), and *related RAS viral* (r-ras) *oncogene homolog 2* (*RRAS2*) and *EPHB1* genes of the mDA axon guidance system were significantly associated with PD. The *DCC rs17468382* and *EPHB1 rs2030737* SNPs seem to be associated with an increased risk of PD, and the *CHP rs6492998* and *RRAS2 rs2970332* SNPs seem to be associated with a reduced risk of PD. Other genetic alterations in Parkinsonian patients may also indirectly induce alterations in axon guidance. Indeed, the LRRK2 protein, encoded by the PARK8 gene, is associated with members of the Disheveled (DVL) family of phosphoproteins, involved in axon guidance and synapse formation. Thus, the PARK8 mutation described in some Parkinsonian patients may indirectly trigger axon guidance failure (Sancho et al., 2009). Finally, it is important to consider that axon-guidance pathway molecules could also contribute to the pathogenesis of PD *via* their roles in axonal repair and in apoptotic signaling. Thus, polymorphisms within the pathway may render neurons more or less vulnerable to endogenous or exogenous toxins that trigger cell death (Shirvan et al., 2000; Yasuhara et al., 2004).

The transcriptional alteration of multiple genes involved in axon guidance has also been described. Using microarrays, Bossers et al. (2009) observed an upregulation by 197% of the repulsive guidance protein RGMA in the SN and caudate putamen of Parkinsonian patients, while the transcript levels of ROBO2 were significantly reduced. In addition, the expression of neuropilin and tolloid (TLL)-like 2 (NETO2) and SLIT and NTRK-like family, member 5 (SLITRK5) that were both decreased in the SN of PD patients. In addition, syndecan 2 (SDC2), a heparan sulfate proteoglycan possibly involved in regulating the function of some axon guidance molecules, was down-regulated in the PD SN. This decreased expression might suggest a shift to a more repulsive nature of the extracellular matrix through a repulsive interaction of SEMA5A with chondroitin sulfate proteoglycans. Indeed, when SEMA5A binds to the heparan sulfate proteoglycan SDC3, it acts as an axon attractant, and when it binds to chondroitin sulfate proteoglycans, it becomes a chemorepulsive protein (Kantor et al., 2004). Altogether, the transcriptional alterations affecting multiple genes involved in axon guidance and neurite outgrowth suggest an altered and possibly more chemorepulsive environment around mDA neurons. Changes in chemorepulsive signaling might contribute to the loss of synaptic contacts between mDA neurons, ultimately leading to a loss in these neurons. Elstner et al. (2009) revealed an upregulation by 1.23 times of the Slit-Robo GTPase activating protein 3 (srGAP3) in dopaminergic neurons of the SNc of PD patients. These results support the importance of the mDA axon guidance systems in the development of PD, because srGAP3 is known to regulate the actin cytosqueleton downstream of Slit-Robo signaling (Bashaw and Klein, 2010).

## Concluding remarks and future directions

Ephrins, Slits, netrins, semaphorins, and Wnt proteins have been described as the main families of axon guidance cues involved in directing mDA axons during development. Most of them have a repulsive effect on the mDA axons except Sema3F, which attracts the mDA axons to the medial prefrontal cortex. It would be of particular interest to investigate whether other attractive cues could be responsible, for instance, for the formation of the tightly fasciculated MFB or for the attraction of the mDA axon terminals to the different tiers of the striatum. In any case, these cells are able to respond to positive or negative environmental signals. Whether their response is sequential or simultaneous to these signals remains to be determined, to better understand how guidance cues can modulate their actions. Indeed, a simultaneous response to opposing signals would result in a push/pull mechanism, defining the precise position of the axon terminals.

Another important point to consider is that mDA axon guidance is often investigated as a homogenous pathway, although it has been described to contain distinct subpopulations of axons that could be differentially guided to their respective targets. It is therefore of critical importance to refine the search for cellular and molecular sources of guidance cues, considering the heterogeneity mDA cell population. This is crucial if the aim is to use these neurons in cell therapy to repair the mDA circuitry in patients with PD. Finally, it appears essential to identify the molecular and cellular environment of grafted cells after transplantation and to determine how these cells are interacting with this molecular environment. This may help to understand how these pathways are anatomically and functionally reconstructed in animal models of PD, and in turn, may improve the efficiency of cell therapy in PD patients.

### Conflict of interest statement

The authors declare that the research was conducted in the absence of any commercial or financial relationships that could be construed as a potential conflict of interest.
